# Global health in the European Union – a review from an agenda-setting perspective

**DOI:** 10.3402/gha.v7.23610

**Published:** 2014-02-13

**Authors:** Christoph Aluttis, Thomas Krafft, Helmut Brand

**Affiliations:** 1 Department of International Health, CAPHRI School for Public Health and Primary Care, Faculty of Health, Medicine and Life Sciences, Maastricht University, Maastricht, The Netherlands; 2 European Academic Global Health Alliance (EAGHA), Brussels, Belgium; 3 Bharati Vidyapeeth Institute of Environment Education and Research, Bharati Vidyapeeth University, Pune, India

**Keywords:** global health, Europe, European Union, agenda setting

## Abstract

This review attempts to analyse the global health agenda-setting process in the European Union (EU). We give an overview of the European perspective on global health, making reference to the developments that led to the EU acknowledging its role as a global health actor. The article thereby focusses in particular on the European interpretation of its role in global health from 2010, which was formalised through, respectively, a European Commission Communication and European Council Conclusions. Departing from there, and based on Kingdon's multiple streams theory on agenda setting, we identify some barriers that seem to hinder the further establishment and promotion of a solid global health agenda in the EU. The main barriers for creating a strong European global health agenda are the fragmentation of the policy community and the lack of a common definition for global health in Europe. Forwarding the agenda in Europe for global health requires more clarification of the common goals and perspectives of the policy community and the use of arising windows of opportunity.

Our health is increasingly shaped by social, economic and environmental factors that are in turn influenced by globalization (1–3). In a globalized and increasingly interdependent world, various European Union (EU) policies are likely to have consequences for health and well-being in other parts of the world (4, 5). However, establishing links between the EU's policies and their health impacts at global scale is a fairly complex task, which has not been consistently taken into consideration in the EU's policies and actions with the third countries (5).

In a response to these claims, more than 300 people came together in Brussels in 2010 to attend a high-level conference on the EU initiative ‘Global health – together we can make it happen’ (6). The conference aimed at discussing the key challenges for achieving good health in a globalised world and sought to develop and refine the European role in this endeavour. The high-level event was organised and hosted by three EU General Directorates (DGs), including the DGs for Research, Development and for Health and Consumers. Participation of these three DGs was attributed to the EU's recognition of global health as a multidisciplinary issue and the EU's commitment to a consolidated approach towards it. Before and during the conference, the central office of the European Commission (EC), the Berlaymont building, was decorated with a banner promoting the EU's commitment to global health. Commentators interpreted this as a strong EU pledge to acting on global health matters, a commitment that had never been ‘so boldly displayed before’ (7). For two days, a wide range of high-level representatives from the EU, the Member States, the World Health Organization (WHO) and other UN organisations, the private sector, academia, and civil society discussed the European role and approach to global health. The conference included the EU's presentation of its communication on ‘The EU role in global health’, which provided a policy framework for the EU's future actions in this field. According to the document, the EU's global health commitment was to focus on four thematic challenges: global health governance, achieving universal health coverage, creating policy coherence, and ensuring that knowledge creation benefits all (8). In his closing speech, former Commissioner for Health and Consumer Policy John Dalli emphasised the willingness to establish a European vision, voice, and action on global health matters, by stating: ‘We are committed to change gear, adjust direction and increase the speed as much as we can to contribute to better health globally. And I can ensure you that the EU will spare no efforts in this quest’ (6). During the conference, it seemed as if a wide range of stakeholders were prepared and willing to enter a policy process, which would contribute to a stronger representation of the EU in a global health context. The EC's communication on the EU role in global health (8), and the corresponding Council Conclusions (9) supported this view. Furthermore, the EC foresaw to publish a European ‘Programme for action’ on global health, which was to define more precisely the activities and actions to be undertaken. But since then, the initiative seems to have lost momentum, which was indicated inter alia by the indefinite postponement of the ‘Programme for action’, as announced during the European Global Health Policy Forum in July 2013 (personal communication, 11 July 2013). This, despite the fact that a global health perspective as a guiding principle for policy has been debated by various national governments across Europe (e.g. in Germany, the United Kingdom, Norway, and Switzerland) as well as in other regions of the world (e.g. Japan and the United States). Also, in academia the discussion on global health and Europe's role in this endeavour is in full swing (10–13). We therefore raise the question of why the global health policy framework has not yet progressed. More specifically, the article has two objectives. In the first part, we will provide an overview of the global health discourse in Europe, highlighting milestones and reflecting on the EU's legitimacy as a global health actor. In the second part, we will build on this review and critically appraise the European global health process after 2010, making use of John W. Kingdon's multiple streams theory (14) to identify issues that influence the agenda-setting processes for global health in the EU.

## The development of a European perspective on global health

While the year 2010 marked an important milestone in the EU's global health commitment, the foundation was laid already in the previous decade. The outbreak of severe acute respiratory syndrome (SARS) in 2003 formed the first global health threat in the new millennium. It forced policy makers in Europe to look beyond their borders in order to protect the health and well-being of their citizen. SARS very vividly illustrated global interdependencies, as the disease spread rapidly via international traffic routes (15). Similarly, the global dimension for health received prominent recognition in the deliberations that led to WHO's Framework Convention on Tobacco Control, an international treaty set up in response to the increasingly global nature of the tobacco industry. This internationally binding treaty represented a novel approach to the global governance of health issues, in which countries showed a willingness to cooperate at a global level in order to effectively address domestic health issues. The increasing recognition of this global dimension for health and the health effects of globalisation also brought the EU to the table. In 2004, former Commissioner for Health and Consumer Protection, David Byrne, expressed the need to link the development of Europe's foreign policy and security identity with health. Byrne believed that health could play a vital role in establishing good relationships between Europe and its global partners (16). The EU's first health strategy, entitled ‘Together for Health’, was adopted in 2007, and it took up the vision expressed by Byrne. The strategy gave direction for the EU's health policy for the period 2008–2013 and included global health as one of four key principles. It stated that ‘in a globalised world, it is hard to separate national or EU-wide actions from the global sphere, as global health issues have an impact on internal community health policy and vice versa’ (4). The strategy highlighted in particular the need for more policy coherence across the EU, coordination with international organisations, and the need to increase the EU's influence and visibility on health matters to ‘match its economic and political weight’ (4). However, these global health priorities were not explicitly included in the European Health Programme 2008–2013, the main instrument of the EC to implement the health strategy. The EC did follow up on the global health principles laid out in the health strategy through different channels. In 2009, the EC's DGs for international development (DG DEV), research (DG RTD), and health and consumers (DG SANCO) decided to join forces and set out to draft the aforementioned communication entitled ‘The EU role in Global Health’. After a formal consultative process with European stakeholders and civil society, the policy framework came to life in 2010. Based on this framework, the Commission defined global health to be about ‘worldwide improvement of health, reduction of disparities, and protection against global health threats.’ Furthermore, the Commission reiterated that ‘addressing global health requires coherence of all internal and external policies and actions based on agreed principles’ (8). The EU's formal acknowledgement that its external policies were not always concerted towards good health outcomes plays an important role in this policy framework, as it explicitly acknowledged the need to extend its ‘health in all policies’ commitment to all its external actions. The EC Communication on its role in global health was further taken up by the Foreign Affairs Council of the EU, which issued their Council Conclusions in response to the EC Communication (9). By this, the Commission Communication received the formal support of the Member States, who reaffirmed the need to act in a consolidated manner on global health issues.

## From policy framework to implementation

In the light of the creation of a policy framework for global health, the EU has been considered to be in a much stronger position to influence related global developments than it was a decade ago. As such, it has been described as an ‘emerging significant player’ (12) and an ‘actor in construction’ (13), with an unfinished agenda for global health (12, 13). Three years after the high-level event and the publication of the EC Communication and Council Conclusion, the European debate seems to have fallen largely silent. Only occasional mentioning of global health can be found in recent EU policy frameworks such as the 3rd European Health Programme 2014–2020, which minimises the global health agenda to the control of cross-border health threats and in particular infectious diseases. The new European research agenda for 2014–2020, entitled ‘Horizon 2020’, also does not give reference to global health as a priority. More generally, EU-funded research focussing on health systems, public health, and the consequences of globalisation on health is likely to be marginalised in the programme (17). Notably, it does acknowledge the need for research partnerships with developing countries and the need for research to achieve the Millennium Development Goals (MDGs), the promotion of HIV/AIDS research and the continuation of the European and Developing Countries Clinical Trial Partnership (EDCTP). However, the European global health agenda initially set out in the EC Communication on its role in global health is much broader than the perspective taken by Horizon 2020.

The absence of a comprehensive global health focus in forward-looking European strategies and actions leads to the conclusion that the EU's global health initiative has lost some of it appeal and is currently not prominently represented on the agenda of the EU. This raises the question of why this is the case.

## Kingdon's multiple streams theory in a European global health context

An important question in European policy making is how and why certain issues make it on the agenda of the EC and how they remain in a prominent position. In our context, the question is why global health was identified as a relevant agenda item that led to the high-level event in 2010, and why it has apparently lost some of its momentum since. To answer this, we will apply Kingdon's theory of multiple streams, which provides a theoretical framework on the question of why certain issues make it on the agenda and others fade. Accordingly, an agenda is defined as ‘the list of subjects to which government officials and those around them are paying serious attention at any given time’ (14). During agenda-setting processes, policy makers narrow down a bulk of possible items to a list that actually becomes the focus of attention. Narrowing down the list of items is of particular relevance to EU policy formulation, because contrary to the popular perception of the EU as an omnipresent bureaucracy, the EU's activities, especially in health, are strictly limited by its mandate and capacity.

**Fig. 1:**
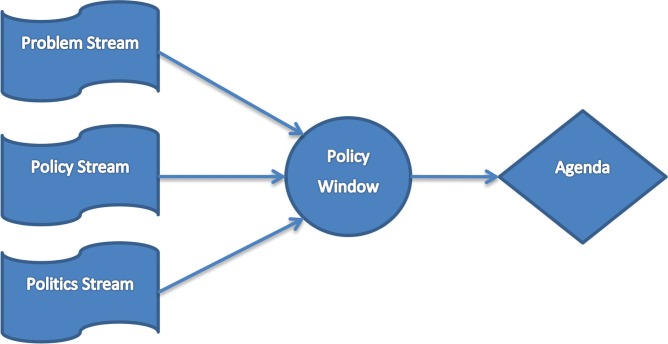
From streams to agenda (based on Kingdon).

At the centre of Kingdon's theory is the assumption that there are three relevant ‘streams’ for agenda setting: problems, policies, and politics (Figure 1). A policy environment (e.g. Brussels) can be viewed as an arena through which these three streams separately and simultaneously surge. The *problem* stream describes ‘those conditions or issues that present themselves as problems, and which require serious attention by policy makers’ (14). It seems only logical that if a problem is identified as such, and communicated effectively to policy makers, its chances to make it on the agenda are significantly enhanced. The *policy* stream, in turn, describes the existence of feasible and acceptable solutions to those problems, developed by specialists in the policy communities in and around Brussels. If feasible and politically acceptable solutions to a problem already exist, the odds for this problem to make it on the agenda of decision makers improve substantially. The third stream, the *politics* stream, includes the macropolitical conditions in a policy environment: the public mood, ideologies of the current leadership, and existence and activities of interest groups and the media (14). All these issues form important promoters or inhibitors for an issue to make it on the agenda.

An issue is most likely to make it on the policy agenda when these three streams come together. This ‘stream convergence’ occurs when a problem is clearly defined, a solution has been developed and is waiting to be implemented, and the public perception for both problems and solutions is favourable. With all three streams aligned, policy makers then anticipate the opening of a ‘policy window’, which creates the opportunity to push the item on the agenda (Figure 1). These windows can open due to both predictable and unpredictable events. Very predictable events include elections and the related changes of personnel at the decision-making level. Turnover of key personnel produces new agenda items as the new people in charge are open to ideas that help them give direction to their leadership. Other policy windows, in turn, are fairly unpredictable. These include the appearance of ‘focussing events’ (usually disasters or crises) that bring everyone's attention to the issue and that can't be ignored. A recent example of this includes the Fukushima nuclear disaster, which moved the Japanese and some European administrations to consider (at least temporally) abandoning their nuclear energy programmes. A further example that illustrates these policy windows was the development of the UK Strategy on Global Health 2008–2013. Problems and solutions for global health had been discussed for several years in the UK policy domain, but only when the SARS crisis hit in 2003, a policy window opened and the UK government took concrete action towards formulating and implementing the UK Strategy on Global Health (18).

## The problem stream for global health in Europe

The problem stream includes the identification and definition of certain issues or conditions as actual problems. Kingdon's theory states that only when there is a clearly identifiable problem, the issue will be taken up on the policy agenda. Problems can be identified via different pathways. Most importantly, the definition and understanding of the actual problem at hand must be clear to all stakeholders. The way that a problem is defined plays a crucial role in agenda-setting processes as, depending on the description of the problem, different solutions will be developed. In a European global health context, this means that the respective issue must be perceived by all stakeholders in the same manner in order to be recognised as a problem. In the European context, however, the policy community working under ‘global health’ terminology is very diverse, and is thereby working according to different problem definitions and interpretations. A discourse on the conceptualisation, scope, and goals of global health is a continuous discussion item among scholars and policy makers, but a clear vision for global health in Europe has been strikingly absent (10). Across Europe, a multitude of different issues labelled as important ‘global health’ problems are put forward. This has been exemplified during the public consultation process that led to the development of the EU Communication on its role in global health (19). According to this process, a majority of European actors interpreted global health as being closely linked to topics of international health and development policy, thereby interpreting it as a normative approach to helping others. In this context, important issues labelled as relevant to global health include the needs to combat HIV/AIDS; ensure food security, adequate sanitation and water supply in third world countries; or reaching the MDGs in general. Other stakeholders, however, interpret the increasing interdependencies and common vulnerabilities that arise due to globalisation as the central theme of global health. Issues identified under this interpretation include inter alia the health effects of climate change, global lifestyle changes, and the impact of global trade on health (19). According to Kingdon, differences in problem formulation create a massive barrier for accurate problem definition and recognition, as different parties have different conceptions of what the global health agenda should consist of. The Commission Communication ultimately tried to short-circuit this debate by setting out in its first paragraph that global health ‘is a term without one single definition’ (8). The decision not to define global health in much detail made its approach acceptable to all stakeholders in Europe. However, this came at the expense of not identifying one, but multiple problems that would need to be tackled. The Communication therefore dilutes its own focus and makes it extremely difficult for policy makers to recognise and define precisely what the ‘problem’ actually is.

## The policy stream for global health in Europe

The lack of a concise and workable definition of global health also affects the policy stream. The policy stream refers to the various ‘solutions’ that have been developed by (European) policy communities in response to certain issues (14). The quality of a particular solution depends on a variety of factors. One key factor is the strength or fragmentation of the policy community that proposes a particular solution. If stakeholders behind a certain issue are very diverse and fragmented in opinion and understanding, the solutions also become fragmented and thereby lose their stability and structure. More closely knit communities, on the other hand, develop common outlooks, orientations, and ways of thinking (14). When policy communities have developed a common language, they can communicate better with each other, and, more importantly, they can communicate more effectively *to others*. This stability allows a solution to be more consistently advocated for. However, in more fragmented communities, instability of issues creates barriers for pressing for the same issue over and over. This seems to be exactly the situation for the European global health community. It appears to be very fragmented in opinion on what solutions should be a priority on the EU's global health agenda. Kingdon notes that policy makers, however, often search for exactly this degree of consensus among organised political forces before deciding to act on an issue (14). This means that if an entire policy community provides policy makers with a powerful impetus to move in a certain direction, policy makers are actually likely to move. But if there is some conflict or disagreement among organised forces, then political leaders implicitly arrive at an image of the issue that depicts it as too difficult to deal with at this particular point in time.

## The political stream and policy windows

Flowing independently of the problem and policy streams is the political stream, which is composed of such things as public mood, ideology, interest group pressure, the media, and other influential actors. Policy makers usually judge whether the general public would tolerate the directions pursued at the political level. Kingdon claims that shifts in this political stream can also be triggered by focussing events (14). In a global health context, the SARS pandemic in 2003 can be interpreted as such a focussing event because it changed the public and political understanding of global interdependencies with a sense of urgency. SARS not only provided an impetus to further coordinate national efforts in cross-border infectious disease control, it also heightened the understanding of a common vulnerability in the face of global health threats (20). Ultimately, SARS acted as a focussing event which not only led to the creation of the European Centre for Disease Control (21) and the revision and update of the International Health Regulations (22), but also led former Commissioner Byrne to push globalisation and health on the EU's agenda, whose high-level support was essential for the process. His emphasis on strengthening Europe's role in global health was announced not only shortly after the time of SARS, but also in the aftermath of the Iraq war, which had led to substantial global divisions politically. In this political mood, Byrne's rationale for a European global health approach would, on the one hand, protect European citizens from infectious diseases, while at the same time act as a driver for peace and stability worldwide (16). His notion to emphasise the benefits to both EU citizens as well as people in third countries allowed for broad stakeholder support. This approach also worked well in the United Kingdom when the government pushed its strategy for global health. Combining domestic health protection with UK leadership in global health governance fell well with both policy makers and the public (18).

However, the awareness for global health and the enthusiasm that led to the EU conference on global health in 2010 have not been preserved since. The European agenda seemed to have quickly given way to other items which were perceived as more pressing. The financial crisis appears to have turned the EU's attention towards economic recovery and enhancing global competitiveness, thereby potentially side-lining the agendas of less influential DGs, such as DG SANCO or DG DEVCO. However, this does not mean that global health is gone for good. According to Kingdon, it is normal that ideas, proposals, or issues rise and fall in favour from time to time. As such, they fade in and fade out, but they never go away (14). The fact that global health is currently not very prominently represented within the EU policy community does therefore not mean that the issue is completely off the European agenda. It merely lies quiet in the community and is being developed further by stakeholders and in academia. Ideas are being sharpened and changed and the longer this process takes, the more people become accustomed to thinking along a global health paradigm. Notably, this so-called preconditioning for global health in Europe is currently taking place and is likely to continue over the coming years, waiting around for additional policy windows to open.

## Summary and conclusion

One of the main insights of the application of Kingdon's theory to global health in a European context is that the large fragmentation of the European global health community is not supportive to pushing global health on the EU agenda. A more coherent understanding and a straightforward conceptualisation of Europe's role in global health would enhance the chances of global health becoming an important agenda item at the European level. Stakeholders for global health need to engage in much more intensive dialogue on the definition and priority areas of a European approach to global health to align their position and to appear to the policy makers as speaking with one voice. In addition, stakeholders and advocates for global health need to continuously work the three streams for global health, so that when a policy window opens, action is more likely to be taken. Early initiatives and think tanks on developing a European perspective on global health have already been established across Europe. Together with research institutions, they need to work on a European conceptualisation of global health.

The elections to the European Parliament and the change of high-level positions within the EC in 2014 will form a policy window to push global health on the agenda again. However, this requires that problems are defined, solutions are available and feasible, and the community has undergone a process of discussion and revision in order to soften up the issue for policy makers and the public. The timely definition of global health and the alignment of the problem, policy, and politics streams are critical to pushing global health back on the agenda of the EC. It should not be forgotten that even in times of economic austerity with its setbacks and hardships, the EU remains a forceful actor in the world which can speak with a strong voice on health matters of global concern.

## References

[1] Huynen MMTE, Martens P, Hilderink H (2005). The health impacts of globalisation: a conceptual framework. Global Health.

[2] Woodward D, Drager N, Beaglehole R, Lipson D (2001). Globalization and health: a framework for analysis and action. Bull World Health Organ.

[3] Dollar D (2001). Is globalization good for your health?. Bull World Health Organ.

[4] European Commission (2007) Together for health: a strategic approach for the EU 2008–2013: COM(2007) 630 final.

[5] Kickbusch I (2006). The need for a European strategy on global health. Scand J Publ Health.

[6] Health & Consumer Voice Conference on Global Health: Together we can make it happen. July 2010. http://ec.europa.eu/dgs/health_consumer/consumervoice/cv_72010_en.pdf.

[7] Global Health Europe (2010). EU event a starting point for a new era in global health governance. http://www.globalhealtheurope.org/index.php/news/81-events/event-reports1/302-eu-event-a-starting-point-for-a-new-era-in-global-health-governance.

[8] European Commission (2010). The EU role in global health: COM(2010)128 final.

[9] Council of the European Union (2010). Council conclusions on the EU role in global health.

[10] Haines A, Flahault A, Horton R (2011). European academic institutions for global health. Lancet.

[11] The Lancet (2012). Europe and global health: looking for a leader. Lancet.

[12] Battams S, van Schaik L, Kickbusch I, Emmerling T The European Union as a Global Health Actor: A critical view.

[13] Rollet V, Chang P (2013). Is the European Union a global health actor? An analysis of its capacities, involvement and challenges. European Foreign Affairs Review.

[14] Kingdon JW (2003). Agendas, alternatives, and public policies.

[15] Ruan S, Wang W, Levin SA (2006). The effect of global travel on the spread of SARS. Math Biosci Eng.

[16] Byrne D (2010). A global health strategy for the European Union. 2004, SPEECH/04/444 during the 7th European Health Forum Gastein.

[17] Walshe K, McKee M, McCarthy M, Groenewegen P, Hansen J, Figueras J (2013). Health systems and policy research in Europe: Horizon 2020. Lancet.

[18] Gagnon ML, Labonte R (2013). Understanding how and why health is integrated into foreign policy – a case study of health is global, a UK Government Strategy 2008–2013. Global Health.

[19] European Commission (2009). Public consultation on the EU role in global health. Summary of contributions.

[20] Liverani M, Coker R (2012). Protecting Europe from diseases: from the international sanitary conferences to the ECDC. J Health Polit Policy Law.

[21] Greer SL (2012). The European Centre for Disease Prevention and Control: hub or hollow core?. J Health Polit Policy Law.

[22] World Health Organization (2005). World health assembly adopts new international health regulations. http://www.who.int/mediacentre/news/releases/2005/pr_wha03/en/.

